# Prior authorization restrictions on medications for opioid use disorder: trends in state laws from 2005 to 2019

**DOI:** 10.1080/07853890.2023.2171107

**Published:** 2023-02-01

**Authors:** Barbara Andraka-Christou, Olivia Golan, Rachel Totaram, Maggie Ohama, Brendan Saloner, Adam J. Gordon, Bradley D. Stein

**Affiliations:** a School of Global Health Management and Informatics, University of Central Florida, Orlando, FL, USA; b Department of Internal Medicine (Secondary Joint Appointment), University of Central Florida, Orlando, FL, USA; c School of Public Health, Georgia State University, Atlanta, GA, USA; d The Cardiac and Vascular Institute, Gainesville, FL, USA; e Bloomberg School of Public Health, Johns Hopkins University, Baltimore, MD, USA; f Informatics, Decision-Enhancement, and Analytic Sciences (IDEAS) Center, VA Salt Lake City Health Care System, Salt Lake City, UT, USA; g Program for Addiction Research, Clinical Care, Knowledge and Advocacy (PARCKA), Division of Epidemiology, Department of Internal Medicine, University of Utah School of Medicine, Salt Lake City, UT, USA; h RAND Corporation, Pittsburgh, PA, USA

**Keywords:** Buprenorphine, methadone, naltrexone, opioid use disorder, medication-assisted treatment, prior authorization, insurance, law, policy

## Abstract

**Research objective:**

Medications for opioid use disorder (MOUDs) – including methadone, buprenorphine, and naltrexone – are the most effective treatments for opioid use disorder (OUD). Historically, insurers have required prior authorization for MOUD, but prior authorization is often reported as a key barrier to MOUD prescribing. Some states have passed laws prohibiting MOUD prior authorization requirements. We sought to identify the frequency of MOUD prior authorization prohibitions in state laws and to categorize types of prohibitions.

**Methods:**

We searched for regulations and statutes present in all U.S. states and Washington DC between 2005 and 2019 using MOUD-related terms in Westlaw legal software. In qualitative software, we coded laws discussing MOUD prior authorization using template analysis – a mixed deductive/inductive approach. Finally, we used coded laws to identify frequencies of states with prior authorization prohibitions, including changes over time.

**Results:**

No states had laws prohibiting MOUD prior authorization between 2005 and 2015, with the first prohibition appearing in 2016. By 2019, fifteen states had MOUD prior authorization prohibitions. States varied significantly in their approach to prohibiting MOUD prior authorization. In 2019, it was more common for states to have MOUD prior authorization prohibitions applying to all insurers (*n* = 10 states) than to only Medicaid (*n* = 7 states) or only non-Medicaid insurers (*n* = 1 state). In 2019, general prior authorization prohibitions (*n* = 10 states) were more common than prohibitions only applicable to medications on the formulary, prohibitions only applicable to medications on the preferred drug list, prohibitions only applicable during the first 5 days of treatment, and prohibitions only applicable during the first 30 days of treatment.

**Conclusions:**

The number of states with an MOUD prior authorization law prohibition increased in recent years. Such laws could help expand access to life-saving OUD treatments by making it easier for clinicians to prescribe MOUD.

**KEY MESSAGES**
- No states had MOUD prior authorization prohibitions between 2005 and 2015 in state statutes or regulations, and only one state had such a prohibition in 2016.
- By 2019, fifteen states had an MOUD prior authorization prohibition law.
- States varied significantly in their approach to prohibiting MOUD prior authorization, including with respect to the insurer type, duration of the prohibition, and applicable medication.

## 1. Introduction

Medications for opioid use disorder (MOUDs) are the gold standard for opioid use disorder (OUD) treatment and include formulations of methadone, buprenorphine, and naltrexone [1]. Methadone and buprenorphine decrease the risk of all-cause mortality for people with OUD by up to 50% [2]. Unfortunately, a recent study found that only approximately 13% of people in the U.S. who could benefit from MOUD receive MOUD [3] and long-term MOUD retention is low [4]. Numerous policy, organizational, clinician, and patient-level barriers exist to MOUD utilization [5–8].

While approximately 10% of buprenorphine prescriptions are paid for out of pocket [9] and others obtain MOUD at no cost (e.g. through syringe service program mobile units distributing free buprenorphine [10]), insurance is a common source of payment for MOUD in the US [11]. Insurers may adopt utilization restrictions, such as prior authorization requirements, for MOUD. Prior authorization refers to the process usually initiated by a health care provider by which a prescription/service must be authorized in advance by the patient’s health plan or insurer [12]. Insurer requirements for prior authorization of MOUD have historically been widespread [12], with prior authorization requirements being one of the most frequently cited buprenorphine prescribing barriers in studies of clinicians [5–8]. Medicaid prior authorization requirements were associated with lowered odds of buprenorphine provision in addiction treatment programs in 2014 and in 2017 [13]. Prior authorization barriers have also been noted in studies regarding naltrexone access [5]. Insurance companies justify prior authorization requirements as limiting unnecessary spending and utilization of medical services, as well as ensuring patient safety [14]. However, in the case of MOUD, prior authorization requirements can cause treatment delays or decreased provision of MOUD [15], putting patients with OUD at risk of poorer clinical outcomes [15].

Most existing reviews of insurance plans’ prior authorization requirements for MOUD focus on Medicaid only [6,12,15–19], with a national study of state Medicaid plans from 2011 through 2013 finding prior authorization for buprenorphine was required in forty-eight states’ Medicaid plans [12]. In contrast, a 2019 study found that only 3% of Medicare plans required prior authorization for buprenorphine treatment of OUD [20]. Commercial insurers appear to have significantly changed buprenorphine prior authorization requirements in recent years [21], with 94% of formularies having at least one buprenorphine product without prior authorization requirements in 2021, as compared to 77% in 2017 [22]. The reasons for such dramatic recent changes in MOUD prior authorization requirements in insurance plans are unknown, including whether changes were mandated by state laws or reflect voluntary action on the part of insurers. It is possible that insurers have increasingly removed MOUD prior authorization requirements in response to state laws, but no study has conducted a comprehensive longitudinal examination of MOUD prior authorization prohibitions in state laws. Information about the legal landscape in which insurers operate is necessary for empirical analyses of policy effects on SUD health service utilization and outcomes.

To address this gap in the literature, we sought to identify the annual prevalence of US states with statutes or regulations that prohibit or otherwise limit prior authorization for methadone, buprenorphine, and/or naltrexone, including within and outside of Medicaid programs, from 2005 to 2019.

## 2. Methods

### 2.1. Data collection

Using search terms related to MOUDs (see Appendix A), we searched for state laws present within 50 US states and the District of Columbia between 2005 and 2019 using Westlaw legal software [23]. Westlaw is a legal database with regulations, statutes, cases, and other policies from U.S. states and territories [23]. Westlaw enables searches of current and historical laws using descriptors and Boolean connectors by jurisdiction [23]. One researcher with legal training then skimmed the text of the Westlaw search results and manually excluded laws that were clearly unrelated to OUD treatment (e.g. medications used solely for pain management), definition sections of laws, and controlled substances schedules. Additional preliminary exclusion criteria are reported elsewhere [24], as this study was part of a larger project exploring legal barriers to MOUD utilization. Remaining state laws were then uploaded into Dedoose [25] qualitative software for further review and analysis. We used the search function in Dedoose to identify laws with the term ‘prior authorization’ (or its derivate terms), and then excluded laws that did not discuss prior authorization in the context of MOUD treatment. For example, laws that reported prior authorization requirements for inpatient substance use disorder treatment, rather than MOUD were excluded at this stage. Finally, since we were most interested in MOUD prior authorization prohibitions, we removed laws that did not prohibit or otherwise restrict prior authorization for MOUD. For example, a law that required prior authorization but did not prohibit prior authorization was removed. See Appendix B for a PRISMA chart of our data collection approach. Institutional review board approval was not sought, as the research did not involve human subjects.

### 2.2. Data analysis

Based on a preliminary review of laws in the final sample, a PhD-level subject matter expert in MOUD policies, who is also an attorney, created an initial codebook in Dedoose software. Using a template analysis approach [26], two researchers independently examined each law, applying the relevant code. Regular meetings were held to discuss any discrepancies in coding and to revise the codebook iteratively to reflect newly identified categories of data.

Three broad categories were included in the codebook: laws applicable to Medicaid only, laws applicable to all insurers in the state, and laws applicable to only non-Medicaid insurance. It was possible for a state to have multiple laws related to prior authorization simultaneously (e.g. one law applicable to Medicaid only and another applicable to non-Medicaid insurers only), especially if two laws were passed subsequently without the first law being repealed. Within each of these broad categories, we then inductively created five sub-categories of MOUD prohibitions appearing in the data: prohibitions only applicable to medication on the formulary; prohibitions only applicable during the first 5 days of treatment; prohibitions only applicable to medication on the preferred drug list; prohibitions only applicable during the first 30 days of treatment; and general prohibitions (i.e. prohibitions not limited to a specific number of days, to the formulary, or to the preferred drug list.) We also created a third level of codes classifying the type of medication maintenance treatment to which the law applied, with options including the following: methadone, buprenorphine, naltrexone, or ‘at least one MOUD’ (i.e. the law bans prior authorization for at least one MOUD without specifying which MOUD).

In those instances where the law broadly referred to MOUD without specifying a type of medication for maintenance treatment, we coded the law as applying to methadone, buprenorphine, and naltrexone. Similarly, if a law applied to at least one MOUD per therapeutic class (i.e. the law prohibits prior authorization for at least one MOUD in each of the agonist, partial agonist, and antagonist classes), then we coded the law as applying to methadone, buprenorphine, and naltrexone for maintenance treatment. We did not code prior authorization prohibitions only applying to methadone dispensed outside of OTPs, because in the US only OTPs may provide long term methadone maintenance treatment for OUD. A spreadsheet was then created and included the following information for each sub-category: the citation of the relevant law, the text of the relevant law, the effective date (identified in Westlaw), and the year(s) in which the law was effective. Two researchers then independently examined the spreadsheet to confirm that the legal text had been categorized correctly. Finally, we conducted basic descriptive statistics to identify the proportion of states with each kind of MOUD prior authorization law and changes in this proportion overtime.

## 3. Results

**Figure 1. States with laws prohibiting prior authorization for MOUD.**
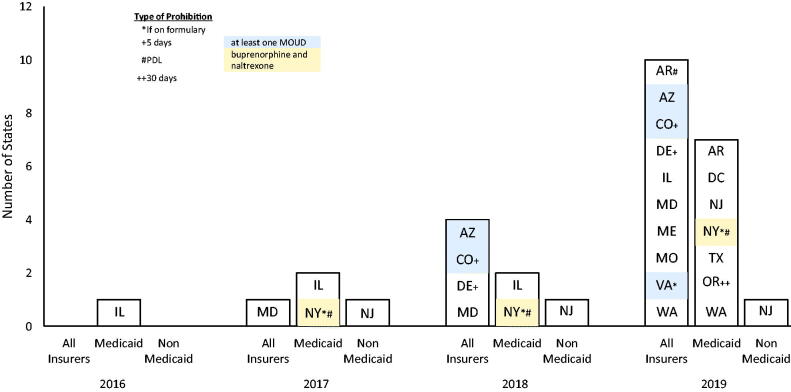


No states had MOUD prior authorization prohibitions in their state statutes or regulations between 2005 and 2015. The number of states with an MOUD prior authorization law prohibition increased from one in 2016 to 15 in 2019. No states repealed MOUD prior authorization prohibitions during the period examined. See Figure 1 for types of prior authorization prohibitions in each state.

In 2019, it was more common for an MOUD prior authorization prohibition to apply to all insurers in the state (*n* = 10 states) than to Medicaid only (*n* = 7 states) or to non-Medicaid insurers only (*n* = 1 state). Among sub-categories of prior authorization prohibitions, general prohibitions were most common. For example, in 2019, ten states had general prior authorization prohibitions, two had prohibitions only applicable to medications on the formulary, two had prohibitions only applicable to medications on the preferred drug list, two had prohibitions only applicable during the first 5 days of treatment, and one had prohibitions only applicable during the first 30 days of treatment. Also, between 2017 and 2019, it was slightly less common for prior authorization prohibitions to apply to methadone maintenance treatment than to buprenorphine or naltrexone. For example, in 2019, while fifteen states had an MOUD prior authorization prohibition, in one of those states the MOUD prior authorization prohibition did not apply to methadone for OUD.

## 4. Discussion

MOUD is a lifesaving treatment for many patients, with buprenorphine and methadone maintenance treatment associated with a 50% reduction in all-cause mortality among people with OUD [2]. Unfortunately, previous studies have indicated clinician concerns about prior authorization [5–8], potentially preventing MOUD prescribing or creating delays in treatment induction [5]. Removal of MOUD prior authorization requirements, whether by government mandate or voluntary insurer action, would likely lead to increased MOUD prescribing among clinicians [27–29] and more timely induction of MOUD, thereby improving health outcomes [28]. This may be particularly true in busy primary care, mental health care, and other high patient volume environments, where taking time to seek prior authorization (a non-reimbursed activity) may be less preferred to using that time to see more patients (a reimbursed activity). For patients, prior authorization delays could also decrease trust in the healthcare system.

We found that the proportion of states with laws prohibiting MOUD prior authorizations has increased dramatically since 2016. Despite this rapid change, in 2019 fewer than a third of states (*n* = 15) had an MOUD prior authorization prohibition. The relatively recent evolution of state prior authorization prohibitions for MOUD may reflect increased policymaker and public attention to the opioid crisis and increasing awareness of the role of insurance prior authorization as a barrier to MOUD treatment. For example, in recent years several popular news media outlets have published stories describing the harms associated with prior authorization requirements for OUD treatment [30,31].

Interestingly, even though we found that only fifteen states had some form of MOUD prior authorization prohibition in 2019, a recent study of commercial insurers found that 80% had stopped requiring prior authorization for immediate-release buprenorphine by 2017 [22]. It remains unknown whether insurers decided to eliminate MOUD prior authorization requirements in their plans in response to state laws. For example, it is possible that insurers changed prior authorization requirements in response to growing use of less-costly generic formulations of buprenorphine [32].

Importantly, we found significant variation across states in the content of laws prohibiting MOUD prior authorization. For example, laws differed with respect to the type of insurer and medication to which the law applied, as well as the duration of the prohibition. Such nuances should be considered in future research examining effects of state policies prohibiting MOUD prior authorization. For example, a blanket prohibition on prior authorization of buprenorphine might have greater implications for MOUD access than a prohibition that only applies to the first 5 days of treatment.

Our results should be considered within the context of our study’s limitations. It is possible that some legal MOUD prior authorization prohibitions do not appear in statutes or regulations. For example, if a Medicaid program released a guideline regarding prior authorization for MOUD, then the guideline might not appear in a legal database like Westlaw. Additionally, our data collection stopped in 2019 and other research suggests that prior authorization policies might have further changed during the COVID-19 pandemic [33]. We did not examine state laws requiring prior authorization, as opposed to prohibiting it, as we believed that individual insurance plans, rather than state laws, would be the most likely source in which mandated prior authorization would appear.

## 5. Conclusion

MOUD is a lifesaving treatment for OUD. Prior authorization can hinder timely access, as it is a known barrier to clinician prescribing of buprenorphine and naltrexone, creating administrative overhead that might not be reimbursable [8,34,35]. By removing this barrier through MOUD prior authorization prohibitions, state laws could facilitate the integration of MOUD prescribing into clinician workflows. We found that state law prohibitions of prior authorization for MOUD are a recent phenomenon, not appearing until 2016. It is unclear whether the recent dramatic decline in the proportion of insurance plans requiring MOUD prior authorization is a response to state laws, lower costs of MOUD, or other factors. Study results could inform future work examining the effects of state prior authorization policies on health service outcomes, such as MOUD utilization. Importantly, prior authorization requirements are only one of many buprenorphine prescribing barriers documented in the literature, including stigma, insufficient staff support, and inadequate clinician training in addiction medicine [5–8]. Nevertheless, state MOUD prior authorization prohibitions indicate some policymakers’ willingness to use legislative and regulatory tools to address buprenorphine prescribing barriers.

## Supplementary Material

Supplemental Material

## Data Availability

State statutes and regulations are publicly available. Our final sample of relevant laws and their citations are available in the Supplemental File.

## Appendix A. Westlaw search strategy

We combined Westlaw search results from the following search strings:

1. ("medication-assisted treatment" OR "medication assisted treatment" OR "medication-assisted treatments" OR "medication assisted treatments" OR (maintenance & opioid!) OR (maintain & opioid!) OR (maintenance & opiate!) OR (maintain & opiate!) OR buprenorphine OR (methadone! & maintain!) OR (methadone! & maintenance) OR (methadone! & addict!) OR (methadone & "use disorder") OR (methadone & "use disorders") OR naltrexone! OR "extended-release naltrexone" OR "extended release naltrexone")
2. (‘prior authorization’ or ‘prior authorize’) AND (agonist or antagonist)

## Appendix B

**PRISMA 2020 flow diagram for new systematic reviews which included searches of databases and registers only**

*From:* Page MJ, McKenzie JE, Bossuyt PM, Boutron I, Hoffmann TC, Mulrow CD, et al. The PRISMA 2020 statement: an updated guideline for reporting systematic reviews. BMJ 2021;372:n71. doi: 10.1136/bmj.n71.

For more information, visit: http://www.prisma-statement.org/

## References

[1] American Society of Addiction Medicine. The ASAM national practice guideline for the treatment of opioid use disorder: 2020 focused update [Internet]. 2020. https://sitefinitystorage.blob.core.windows.net/sitefinity-production-blobs/docs/default-source/guidelines/npg-jam-supplement.pdf?sfvrsn=a00a52c2_2. 10.1097/ADM.0000000000000633 32511106

[2] Santo T, Jr., Clark B, Hickman M, et al. Association of opioid agonist treatment with All-Cause mortality and specific causes of death among people with opioid dependence: a systematic review and meta-analysis. JAMA Psychiatry. 2021;78(9):979–993. 34076676 10.1001/jamapsychiatry.2021.0976 PMC8173472

[3] Krawczyk N, Rivera BD, Jent V, et al. Has the treatment gap for opioid use disorder narrowed in the U.S.?: a yearly assessment from 2010 to 2019. Int J Drug Policy. 2022;110:103786. 35934583 10.1016/j.drugpo.2022.103786 PMC10976290

[4] O’Connor AM, Cousins G, Durand L, et al. Retention of patients in opioid substitution treatment: a systematic review. PLoS One. 2020;15(5):e0232086. 32407321 10.1371/journal.pone.0232086 PMC7224511

[5] Andraka-Christou B, Capone MJ. A qualitative study comparing physician-reported barriers to treating addiction using buprenorphine and extended-release naltrexone in U.S. office-based practices. Int J Drug Policy. 2018;54:9–17. 29324253 10.1016/j.drugpo.2017.11.021

[6] GAO. Opioid use disorder: barriers to Medicaid beneficiaries’ access to treatment medications. Report to Congressional Committees; 2020.

[7] Haffajee RL, Andraka-Christou B, Attermann J, et al. A mixed-method comparison of physician-reported beliefs about and barriers to treatment with medications for opioid use disorder. Subst Abuse Treat Prev Policy. 2020;15(1):69. 32928272 10.1186/s13011-020-00312-3 PMC7491096

[8] Jones CM, McCance-Katz EF. Characteristics and prescribing practices of clinicians recently waivered to prescribe buprenorphine for the treatment of opioid use disorder. Addiction. 2019;114(3):471–482. 30194876 10.1111/add.14436

[9] Stein BD, Jones CM, Smart R, et al. Patient, prescriber, and community factors associated with filled naloxone prescriptions among patients receiving buprenorphine 2017-18. Drug Alcohol Depend. 2021;221:108569. 33578296 10.1016/j.drugalcdep.2021.108569 PMC8027950

[10] Krawczyk N, Buresh M, Gordon MS, et al. Expanding low-threshold buprenorphine to justice-involved individuals through mobile treatment: addressing a critical care gap. J Subst Abuse Treat. 2019;103:1–8. 31229187 10.1016/j.jsat.2019.05.002 PMC6612429

[11] Nguyen TD, Gupta S, Ziedan E, et al. Assessment of filled buprenorphine prescriptions for opioid use disorder during the coronavirus disease 2019 pandemic. JAMA Intern Med. 2021;181(4):562–565. 33346795 10.1001/jamainternmed.2020.7497 PMC7754073

[12] SAMHSA. Medicaid coverage of medication-assisted treatment for alcohol and opioid use disorders and of medication for the reversal of opioid overdose. 2018.

[13] Andrews CMA, Grogan AJ, Westlake CM, et al. Impact of Medicaid restrictions on availability of buprenorphine in addiction treatment programs. Am J Public Health. 2019;109(3):434–436. 30676789 10.2105/AJPH.2018.304856 PMC6366513

[14] Bachhuber MA. How do state Medicaid programs determine what substance use disorder treatment medications need prior authorization? An overview for clinicians. Addict Sci Clin Pract. 2020;15(1):20. 32600402 10.1186/s13722-020-00194-7 PMC7325260

[15] ASAM. Advancing access to addiction medications: implications for opioid addiction treatment. Rockville (MD): ASAM; 2013.

[16] DPA. With goal of reducing overdose deaths, New Jersey Assembly Committee votes to end prior authorization for medication assisted treatment under Medicaid. New York: Drug Policy Alliance; 2019.

[17] Macpac. Report to congress on Medicaid and CHIP [Internet]. 2017. https://www.macpac.gov/publication/june-2017-report-to-congress-on-medicaid-and-chip/.

[18] Mark TL, Lubran R, McCance-Katz EF, et al. Medicaid Coverage of medications to treat alcohol and opioid dependence. J Subst Abuse Treat. 2015;55:1–5. 25921475 10.1016/j.jsat.2015.04.009

[19] SAMHSA. Medicaid coverage and financing of medications to treat alcohol and opioid use disorders. Rockville (MD): Substance Abuse and Mental Health Services Administration; 2014.

[20] Mark TL, Parish WJ, Weber EM, et al. Prior authorization for opioid use disorder versus pain medications: lessons learned for parity enforcement. J Stud Alcohol Drugs. 2021;82(2):214–218. 33823968

[21] Reif S, Creedon TB, Horgan CM, et al. Commercial health plan coverage of selected treatments for opioid use disorders from 2003 to 2014. J Psychoactive Drugs. 2017;49(2):102–110. 28350229 10.1080/02791072.2017.1300360 PMC5861719

[22] Nguyen TD, Chua K-P, Andraka-Christou B, et al. Trends in buprenorphine coverage and prior authorization requirements in US commercial formularies, 2017-2021. JAMA Health Forum. 2022;3(7):e221821. 35977219 10.1001/jamahealthforum.2022.1821 PMC9270692

[23] Westlaw. Toronto: Thompson Reuters; 2022.

[24] Andraka-Christou B, Gordon AJ, Bouskill K, et al. Toward a typology of office-based buprenorphine treatment laws: themes from a review of state laws. J Addict Med. 2022;16(2):192–207. 34014209 10.1097/ADM.0000000000000863 PMC8599526

[25] Dedoose. Version 8.0.35. 2018. www.dedoose.com.

[26] Brooks J, McCluskey S, Turley E, et al. The Utility of template analysis in qualitative psychology research. Qual Res Psychol. 2015;12(2):202–222. 27499705 10.1080/14780887.2014.955224 PMC4960514

[27] Ferries E, Racsa P, Bizzell B, et al. Removal of prior authorization for medication-assisted treatment: impact on opioid use and policy implications in a Medicare advantage population. J Manag Care Spec Pharm. 2021;27(5):596–606. 33908274 10.18553/jmcp.2021.27.5.596 PMC10390915

[28] Mark TL, Parish WJ, Zarkin GA. Association of formulary prior authorization policies with buprenorphine-naloxone prescriptions and hospital and emergency department use among Medicare beneficiaries. JAMA Netw Open. 2020;3(4):e203132. 32310285 10.1001/jamanetworkopen.2020.3132 PMC7171554

[29] Parish WJ, Mark TL, Zarkin GA, et al. The association of Medicare part D prior authorization for buprenorphine-naloxone with adherence to opioid use disorder treatment guidelines in the United States. Addiction. 2022;117(1):141–150. 34033177 10.1111/add.15585

[30] Goodnough A. The Treatment gap: when an Iowa family doctor takes on the opioid epidemic. The New York Times [Internet]. 2018 Jun 23. [cited 2022 Jul 27]. https://www.nytimes.com/2018/06/23/health/opioid-addiction-suboxone-treatment.html

[31] Harper J. Insurance rules can hamper recovery from opioid addiction. National Public Radio [Internet]. 2016 Aug 5. [cited 2022 Jul 27]. https://www.npr.org/sections/health-shots/2016/08/05/485554456/insurance-rules-can-hamper-recovery-from-opioid-addiction

[32] Roberts AW, Saloner B, Dusetzina SB. Buprenorphine use and spending for opioid use disorder treatment: trends from 2003 to 2015. Psychiatr Serv. 2018;69(7):832–835. 29734918 10.1176/appi.ps.201700315 PMC6028283

[33] Andraka-Christou B, Bouskill K, Haffajee RL, et al. Common themes in early state policy responses to substance use disorder treatment during COVID-19. Am J Drug Alcohol Abuse. 2021;47(4):486–496. 33909518 10.1080/00952990.2021.1903023 PMC8564552

[34] Andrilla CHA, Jones KC, Patterson DG. Prescribing practices of nurse practitioners and physician assistants waivered to prescribe buprenorphine and the barriers they experience prescribing buprenorphine. J Rural Health. 2020;36(2):187–195. 31650634 10.1111/jrh.12404

[35] Beetham T, Saloner B, Wakeman SE, et al. Access to office-based buprenorphine treatment in areas with high rates of opioid-related mortality: an audit study. Ann Intern Med. 2019;171(1):1–9. 31158849 10.7326/M18-3457 PMC7164610

