# Supplementary material for: Prior authorization restrictions on medications for opioid use disorder: trends in state laws from 2005 to 2019
Source: Ann Med. 2023 Feb 1;55(1):514–20. doi: 10.1080/07853890.2023.2171107 (PMC9897778; doi:10.1080/07853890.2023.2171107)
Supplement: Supplemental Material [file IANN_A_2171107_SM0970.docx]

**SUPPLEMENTAL FILE: STATE STATUTES & REGULATIONS WITH PRIOR AUTHORIZATION PROHIBITIONS**

| State | Citation | Excerpt | Payer | Medication | Type of Prohibition | Year |
| --- | --- | --- | --- | --- | --- | --- |
| CO | CO 2019 § 10-16-104. Effective January 1, 2019. | (5.5)(a)(III)(B) A health benefit plan subject to this subsection (5.5) must provide coverage without prior authorization for a five-day supply of at least one of the federal food and drug administration-approved drugs for the treatment of opioid dependence; except that this requirement is limited to a first request within a twelve-month period.   CO 2019 702-4:4-2-49. Amended regulation effective October 1, 2019  All carriers issuing individual and group health benefit plans shall make available and provide coverage for, without prior authorization, a five (5) day supply of at least one (1) of the FDA-approved drugs prescribed for the treatment of opioid dependence. This requirement is limited to a first request within a twelve (12) month period. | All insurers | At least one MOUD | 5 days | 2019 |
| CO | CO 2018 702-4:4-2-49. Amended Jan. 1, 2019. | A. All carriers issuing individual and group health benefit plans shall: 1. Make available and provide coverage for, without prior authorization, a five (5) day supply of at least one (1) of the federal Food and Drug Administration-approved drugs prescribed for the treatment of opioid dependence. This requirement is limited to a first request within a twelve (12) month period; | All insurers | At least one MOUD | 5 days | 2018 |
| AZ | AZ 2019 A.R.S. § 20-3402. Effective: April 26, 2018. | B. A health care services plan must allow at least one modality of medication-assisted treatment to be available without prior authorization. | All insurers | At least one MOUD | General | 2019 |
| AZ | AZ 2018 A.R.S. § 20-3402. Effective: April 26, 2018. | B. A health care services plan must allow at least one modality of medication-assisted treatment to be available without prior authorization. | All insurers | At least one MOUD | General | 2018 |
| VA | VA 2019 § 38.2-3407.15:2. Effective: July 1, 2019. | 12. Require that no prior authorization be required for at least one drug prescribed for substance abuse medication-assisted treatment, provided that (i) the drug is a covered benefit, (ii) the prescription does not exceed the FDA labeled dosages, and (iii) the drug is prescribed consistent with the regulations of the Board of Medicine. | All insurers | At least one MOUD | If on formulary | 2019 |
| ME | ME 2019 24-A M.R.S.A. § 4304. Effective: September 19, 2019. | 2-A. Prior authorization of medication-assisted treatment for opioid use disorder. A carrier may not require prior authorization for medication-assisted treatment for opioid use disorder for the prescription of at least one drug for each therapeutic class of medication used in medication-assisted treatment, except that a carrier may not impose any prior authorization requirements on a pregnant woman for medication-assisted treatment for opioid use disorder. For the purposes of this subsection, “medication-assisted treatment” means an evidence-based practice that combines pharmacological interventions with substance use disorder counseling. | All insurers | at least one MOUD per class | General | 2019 |
| WA | WA 2019 48.43.760. Effective July 28, 2019. | For health plans issued or renewed on or after January 1, 2020, a health carrier shall provide coverage without prior authorization of at least one federal food and drug administration approved product for the treatment of opioid use disorder in the drug classes opioid agonists, opioid antagonists, and opioid partial agonists. | All insurers | At least one MOUD per class | General | 2019 |
| WA | WA 2019 74.09.645. Effective: July 28, 2019. | Upon initiation or renewal of a contract with the authority to administer a medicaid managed care plan, a managed health care system shall provide coverage without prior authorization of at least one federal food and drug administration approved product for the treatment of opioid use disorder in the drug classes opioid agonists, opioid antagonists, and opioid partial agonists. | Medicaid | at least one MOUD per class | General | 2019 |
| OR | O.R.S. § Ch. 583, § 7 (eff. July 23, 2019) | Ch. 583, § 7. Restrictions on prior authorization requirements for medication-assisted treatment of substance abuse disorders; rulemaking authority  (1) The Oregon Health Authority shall prohibit coordinated care organizations and public payers of health insurance, when reimbursing the cost of medication-assisted treatment for treating substance use disorders, including opioid and opiate addiction, from requiring prior authorization of payment during the first 30 days of medication-assisted treatment. (2) The authority may adopt rules to carry out this section. Credits Laws 2019, c. 583, § 7, eff. July 23, 2019, operative Jan. 1, 2020. | Medicaid | buprenorphine | 30 days | 2019 |
| AR | AR 2019 A.C.A. § 23-99-1119 Effective: April 12, 2019 | (a) Except in the case of injectables, a healthcare insurer, including Medicaid, shall not: (1) Require prior authorization in order for a patient to obtain coverage of buprenorphine, naloxone, naltrexone, methadone, and their various formulations and combinations approved by the United States Food and Drug Administration for the treatment of opioid addiction | All insurers | buprenorphine | General | 2019 |
| DC | DC 2019 § 31-3175.05 Effective: March 13, 2019 | Medicaid shall provide coverage for medication-assisted treatment prescribed for the treatment of substance use disorders; provided, that medication assisted treatment covered in accordance with this section shall not be subject to:   (1) Utilization control, other than those processes specified by the American Society of Addiction Medicine;   (2) Prior authorization; | Medicaid | buprenorphine | General | 2019 |
| IL | IL 2019 215 ILCS 5/370c Effective: August 16, 2019 | (6) An issuer of a group health benefit plan may provide or offer coverage required under this Section through a managed care plan. (6.5) An individual or group health benefit plan amended, delivered, issued, or renewed on or after January 1, 2019 (the effective date of Public Act 100-1024): (A) shall not impose prior authorization requirements, other than those established under the Treatment Criteria for Addictive, Substance-Related, and Co-Occurring Conditions established by the American Society of Addiction Medicine, on a prescription medication approved by the United States Food and Drug Administration that is prescribed or administered for the treatment of substance use disorders; | All insurers | buprenorphine | General | 2019 |
| MD | MD 2019 § 15-851 Effective: May 25, 2017 | § 15-851. Prior authorization requirement for drugs to treat opioid use disorders prohibited Currentness Scope of section (a)(1) This section applies to: (i) insurers and nonprofit health service plans that provide coverage for substance use disorder benefits or prescription drugs under individual, group, or blanket health insurance policies or contracts that are issued or delivered in the State; and (ii) health maintenance organizations that provide coverage for substance use disorder benefits or prescription drugs under individual or group contracts that are issued or delivered in the State. (2) An insurer, a nonprofit health service plan, or a health maintenance organization that provides coverage for substance use disorder benefits under the medical benefit or for prescription drugs through a pharmacy benefits manager is subject to the requirements of this section. Prohibition (b) An entity subject to this section may not apply a prior authorization requirement for a prescription drug: (1) when used for treatment of an opioid use disorder; and (2) that contains methadone, buprenorphine, or naltrexone. | All insurers | buprenorphine | General | 2019 |
| MO | MO 2019 V.A.M.S. 191.1165 Effective: August 28, 2019 | MO 2019 V.A.M.S. 191.1165 Effective: August 28, 2019 3. MAT medications provided for in this section shall not be subject to any of the following: (1) Any annual or lifetime dollar limitations; (2) Financial requirements and quantitative treatment limitations that do not comply with the Mental Health Parity and Addiction Equity Act of 2008 (MHPAEA), specifically 45 CFR 146.136(c)(3); (3) Step therapy or other similar drug utilization strategy or policy when it conflicts or interferes with a prescribed or recommended course of treatment from a licensed health care professional; and (4) Prior authorization for MAT medications as specified in this section. 4. MAT medications outlined in this section shall apply to all health insurance plans delivered in the state of Missouri. | All insurers | buprenorphine | General | 2019 |
| NJ | NJ 2019 30:4D-6m <Section effective on Oct. 13, 2019.> | Provision of benefits for certain drugs used in the treatment of substance use disorders; guidelines for safety and efficacy Currentness <Section effective on Oct. 13, 2019.> a. Notwithstanding any State law or regulation to the contrary, the Department of Human Services shall ensure that the provision of benefits for methadone, buprenorphine, naltrexone, combination drugs containing buprenorphine and naloxone, and, if authorized by the Commissioner of Human Services, other medications approved by the federal Food and Drug Administration for the treatment of substance use disorders, to eligible persons receiving services funded by the Division of Mental Health and Addiction Services in the Department of Human Services as well as to eligible persons under the Medicaid program, established pursuant to P.L.1968, c. 413 (C.30:4D-1 et seq.), shall be provided without the imposition of any prior authorization requirements, provided that the treatment is prescribed or administered by a licensed medical practitioner who is authorized to prescribe or administer that treatment pursuant to State and federal law. The Commissioner of Human Services may develop and issue guidelines to ensure the safety and efficacy of benefits provided pursuant to this section. b. As used in this act: “Substance use disorder” is as defined by the American Psychiatric Association in the Diagnostic and Statistical Manual of Mental Disorders, Fifth Edition and any subsequent editions and shall include substance use withdrawal. | Medicaid | buprenorphine | General | 2019 |
| NJ | NJ 2019 1748A-7kk eff. May 16, 2017 | i. The benefits for medication-assisted treatments for substance use disorder shall be provided when determined medically necessary by the covered person's physician, psychologist or psychiatrist without the imposition of any prior authorization or other prospective utilization management requirements. | Non Medicaid | buprenorphine | General | 2019 |
| TX | TX 2019 § 32.03115 Effective: September 1, 2019 | (a) In this section, “medication-assisted opioid or substance use disorder treatment” means the use of methadone, buprenorphine, oral buprenorphine/naloxone, or naltrexone to treat opioid or substance use disorder. (b) Notwithstanding Sections 531.072 and 531.073, Government Code, or any other law and subject to Subsections (c) and (d), the commission shall provide medical assistance reimbursement for medication-assisted opioid or substance use disorder treatment without requiring a recipient of medical assistance or health care provider to obtain prior authorization or precertification for the treatment, except as needed to minimize the opportunity for fraud, waste, or abuse.1 (c) The duty to provide medical assistance reimbursement for medication-assisted opioid or substance use disorder treatment under Subsection (b) does not apply with respect to: (1) a prescription for methadone; | Medicaid | buprenorphine | General | 2019 |
| IL | IL 2018 305 ILCS 5/5-5 Effective: January 1, 2018 | Notwithstanding any other provision of this Code to the contrary, on or after July 1, 2015, all FDA approved forms of medication assisted treatment prescribed for the treatment of alcohol dependence or treatment of opioid dependence shall be covered under both fee for service and managed care medical assistance programs for persons who are otherwise eligible for medical assistance under this Article and shall not be subject to any (1) utilization control, other than those established under the American Society of Addiction Medicine patient placement criteria, (2) prior authorization mandate, or (3) lifetime restriction limit mandate. On or after July 1, 2015, opioid antagonists prescribed for the treatment of an opioid overdose, including the medication product, administration devices, and any pharmacy fees related to the dispensing and administration of the opioid antagonist, shall be covered under the medical assistance program for persons who are otherwise eligible for medical assistance under this Article. As used in this Section, “opioid antagonist” means a drug that binds to opioid receptors and blocks or inhibits the effect of opioids acting on those receptors, including, but not limited to, naloxone hydrochloride or any other similarly acting drug approved by the U.S. Food and Drug Administration. | Medicaid | buprenorphine | General | 2018 |
| MD | MD 2018 § 15-851 Effective: May 25, 2017 | § 15-851. Prior authorization requirement for drugs to treat opioid use disorders prohibited Currentness Scope of section (a)(1) This section applies to: (i) insurers and nonprofit health service plans that provide coverage for substance use disorder benefits or prescription drugs under individual, group, or blanket health insurance policies or contracts that are issued or delivered in the State; and (ii) health maintenance organizations that provide coverage for substance use disorder benefits or prescription drugs under individual or group contracts that are issued or delivered in the State. (2) An insurer, a nonprofit health service plan, or a health maintenance organization that provides coverage for substance use disorder benefits under the medical benefit or for prescription drugs through a pharmacy benefits manager is subject to the requirements of this section. Prohibition (b) An entity subject to this section may not apply a prior authorization requirement for a prescription drug: (1) when used for treatment of an opioid use disorder; and (2) that contains methadone, buprenorphine, or naltrexone. Credits Added by Acts 2017, c. 581, § 1, eff. May 25, 2017. | All insurers | buprenorphine | General | 2018 |
| NJ | NJ 2018 17:48A-7kk eff. May 16, 2017 | i. The benefits for medication-assisted treatments for substance use disorder shall be provided when determined medically necessary by the covered person's physician, psychologist or psychiatrist without the imposition of any prior authorization or other prospective utilization management requirements. | NonMedicaid | buprenorphine | General | 2018 |
| IL | IL 2017 305 ILCS 5/5-5 Effective: January 1, 2017 | Notwithstanding any other provision of this Code to the contrary, on or after July 1, 2015, all FDA approved forms of medication assisted treatment prescribed for the treatment of alcohol dependence or treatment of opioid dependence shall be covered under both fee for service and managed care medical assistance programs for persons who are otherwise eligible for medical assistance under this Article and shall not be subject to any (1) utilization control, other than those established under the American Society of Addiction Medicine patient placement criteria, (2) prior authorization mandate, or (3) lifetime restriction limit mandate. On or after July 1, 2015, opioid antagonists prescribed for the treatment of an opioid overdose, including the medication product, administration devices, and any pharmacy fees related to the dispensing and administration of the opioid antagonist, shall be covered under the medical assistance program for persons who are otherwise eligible for medical assistance under this Article. As used in this Section, “opioid antagonist” means a drug that binds to opioid receptors and blocks or inhibits the effect of opioids acting on those receptors, including, but not limited to, naloxone hydrochloride or any other similarly acting drug approved by the U.S. Food and Drug Administration. | Medicaid | buprenorphine | General | 2017 |
| MD | MD 2017 § 15-851 Effective: May 25, 2017 | MD Code, Insurance, § 15-851 § 15-851. Prior authorization requirement for drugs to treat opioid use disorders prohibited Currentness Scope of section (a)(1) This section applies to: (i) insurers and nonprofit health service plans that provide coverage for substance use disorder benefits or prescription drugs under individual, group, or blanket health insurance policies or contracts that are issued or delivered in the State; and (ii) health maintenance organizations that provide coverage for substance use disorder benefits or prescription drugs under individual or group contracts that are issued or delivered in the State. (2) An insurer, a nonprofit health service plan, or a health maintenance organization that provides coverage for substance use disorder benefits under the medical benefit or for prescription drugs through a pharmacy benefits manager is subject to the requirements of this section. Prohibition (b) An entity subject to this section may not apply a prior authorization requirement for a prescription drug: (1) when used for treatment of an opioid use disorder; and (2) that contains methadone, buprenorphine, or naltrexone. | All insurers | buprenorphine | General | 2017 |
| NJ | NJ 2017 17:48A-7kk Effective on May 16, 2017 | i. The benefits for medication-assisted treatments for substance use disorder shall be provided when determined medically necessary by the covered person's physician, psychologist or psychiatrist without the imposition of any prior authorization or other prospective utilization management requirements. | NonMedicaid | buprenorphine | General | 2017 |
| IL | IL 2016 305 ILCS 5/5-5 Effective: July 1, 2016 | Notwithstanding any other provision of this Code to the contrary, on or after July 1, 2015, all FDA approved forms of medication assisted treatment prescribed for the treatment of alcohol dependence or treatment of opioid dependence shall be covered under both fee for service and managed care medical assistance programs for persons who are otherwise eligible for medical assistance under this Article and shall not be subject to any (1) utilization control, other than those established under the American Society of Addiction Medicine patient placement criteria, (2) prior authorization mandate, or (3) lifetime restriction limit mandate.  2nd law: IL 2016 305 ILCS 5/5-5 September 9, 2015 to June 30, 2016 Notwithstanding any other provision of this Code to the contrary, on or after July 1, 2015, all FDA approved forms of medication assisted treatment prescribed for the treatment of alcohol dependence or treatment of opioid dependence shall be covered under both fee for service and managed care medical assistance programs for persons who are otherwise eligible for medical assistance under this Article and shall not be subject to any (1) utilization control, other than those established under the American Society of Addiction Medicine patient placement criteria, (2) prior authorization mandate, or (3) lifetime restriction limit mandate. On or after July 1, 2015, opioid antagonists prescribed for the treatment of an opioid overdose, including the medication product, administration devices, and any pharmacy fees related to the dispensing and administration of the opioid antagonist, shall be covered under the medical assistance program for persons who are otherwise eligible for medical assistance under this Article. As used in this Section, “opioid antagonist” means a drug that binds to opioid receptors and blocks or inhibits the effect of opioids acting on those receptors, including, but not limited to, naloxone hydrochloride or any other similarly acting drug approved by the U.S. Food and Drug Administration. | Medicaid | buprenorphine | General | 2016 |
| NY | NY 2019 § 273. Effective: June 22, 2016. | 10. Prior authorization shall not be required for an initial or renewal prescription for buprenorphine or injectable naltrexone for detoxification or maintenance treatment of opioid addiction unless the prescription is for a non-preferred or non-formulary form of such drug as otherwise required by section 1927(k)(6) of the Social Security Act. | Medicaid | buprenorphine | If on formulary | 2019 |
| NY | NY 2019 § 364-j. Effective: December 16, 2019. | 26-b. Managed care providers shall not require prior authorization for an initial or renewal prescription for buprenorphine or injectable naltrexone for detoxification or maintenance treatment of opioid addiction unless the prescription is for a non-preferred or non-formulary form of the drug or as otherwise required by section 1927(k)(6) of the Social Security Act. | Medicaid | buprenorphine | If on formulary | 2019 |
| NY | NY 2018 § 273. Effective: June 22, 2016. | 10. Prior authorization shall not be required for an initial or renewal prescription for buprenorphine or injectable naltrexone for detoxification or maintenance treatment of opioid addiction unless the prescription is for a non-preferred or non-formulary form of such drug as otherwise required by section 1927(k)(6) of the Social Security Act. | Medicaid | buprenorphine | If on formulary | 2018 |
| NY | NY 2018 § 273. Effective: June 22, 2016. | 10. Prior authorization shall not be required for an initial or renewal prescription for buprenorphine or injectable naltrexone for detoxification or maintenance treatment of opioid addiction unless the prescription is for a non-preferred or non-formulary form of such drug as otherwise required by section 1927(k)(6) of the Social Security Act. | Medicaid | buprenorphine | If on formulary | 2018 |
| NY | NY 2017 § 273. Effective: June 22, 2016. | 10. Prior authorization shall not be required for an initial or renewal prescription for buprenorphine or injectable naltrexone for detoxification or maintenance treatment of opioid addiction unless the prescription is for a non-preferred or non-formulary form of such drug as otherwise required by section 1927(k)(6) of the Social Security Act. | Medicaid | buprenorphine | If on formulary | 2017 |
| NY | NY 2017 § 273. Effective: June 22, 2016. | 10. Prior authorization shall not be required for an initial or renewal prescription for buprenorphine or injectable naltrexone for detoxification or maintenance treatment of opioid addiction unless the prescription is for a non-preferred or non-formulary form of such drug as otherwise required by section 1927(k)(6) of the Social Security Act. | Medicaid | buprenorphine | If on formulary | 2017 |
| DE | DE 2019 § 3343. eff. Aug. 29, 2018. | (2) a. A health benefit plan that provides coverage for prescription drugs must provide coverage for the treatment of serious mental illnesses and drug and alcohol dependencies that includes immediate access, without prior authorization, to a 5-day emergency supply of prescribed medications covered under the health benefit plan for the medically necessary treatment of serious mental illnesses and drug and alcohol dependencies where an emergency medical condition, as defined in § 3349(e) of this title, exists, including a prescribed drug or medication associated with the management of opioid withdrawal or stabilization, except where otherwise prohibited by law.  DE 2019 § 3578. eff. Sept. 29, 2017.  (2) a. A health benefit plan that provides coverage for prescription drugs must provide coverage for the treatment of serious mental illnesses and drug and alcohol dependencies that include immediate access, without prior authorization, to a 5 day emergency supply of prescribed medications covered under the health benefit plan for the medically necessary treatment of serious mental illnesses and drug and alcohol dependencies where an emergency medical condition, as defined in § 3565(e) of this title, exists, including a prescribed drug or medication associated with the management of opioid withdrawal or stabilization, except where otherwise prohibited by law. | All insurers | buprenorphine | If on formulary, 5 days | 2019 |
| DE | DE 2018 § 3343. eff. Aug. 29, 2018. | (2) a. A health benefit plan that provides coverage for prescription drugs must provide coverage for the treatment of serious mental illnesses and drug and alcohol dependencies that includes immediate access, without prior authorization, to a 5-day emergency supply of prescribed medications covered under the health benefit plan for the medically necessary treatment of serious mental illnesses and drug and alcohol dependencies where an emergency medical condition, as defined in § 3349(e) of this title, exists, including a prescribed drug or medication associated with the management of opioid withdrawal or stabilization, except where otherwise prohibited by law.  DE 2018 § 3578. eff. Sept. 29, 2017.  (2) a. A health benefit plan that provides coverage for prescription drugs must provide coverage for the treatment of serious mental illnesses and drug and alcohol dependencies that include immediate access, without prior authorization, to a 5 day emergency supply of prescribed medications covered under the health benefit plan for the medically necessary treatment of serious mental illnesses and drug and alcohol dependencies where an emergency medical condition, as defined in § 3565(e) of this title, exists, including a prescribed drug or medication associated with the management of opioid withdrawal or stabilization, except where otherwise prohibited by law. | All insurers | buprenorphine | If on formulary, 5 days | 2018 |
| AR | AR 2019 A.C.A. § 23-99-1119 - Effective: April 12, 2019. | (a) Except in the case of injectables, a healthcare insurer, including Medicaid, shall not: (1) Require prior authorization in order for a patient to obtain coverage of buprenorphine, naloxone, naltrexone, methadone, and their various formulations and combinations approved by the United States Food and Drug Administration for the treatment of opioid addiction; or (2) Impose any other requirement other than a valid prescription and compliance with the medication-assisted treatment guidelines issued by the Substance Abuse and Mental Health Services Administration under the United States Department of Health and Human Services in order for a patient to obtain coverage for buprenorphine, naloxone, naltrexone, methadone, and their various formulations and combinations approved by the United States Food and Drug Administration for the treatment of opioid addiction. (b) Subdivision (a)(1) of this section shall only apply to the Arkansas Medicaid Program as it pertains to prescription drugs for treatment of opioid addiction designated as preferred on the evidence-based preferred drug list provided there is at least one (1) of each of the drugs listed in subdivision (a)(1) of this section with the preferred designation on the preferred drug list or available without prior authorization. | Medicaid | buprenorphine | PDL | 2019 |
| NY | NY 2019 § 273. Effective: June 22, 2016 | 10. Prior authorization shall not be required for an initial or renewal prescription for buprenorphine or injectable naltrexone for detoxification or maintenance treatment of opioid addiction unless the prescription is for a non-preferred or non-formulary form of such drug as otherwise required by section 1927(k)(6) of the Social Security Act. | Medicaid | buprenorphine | PDL | 2019 |
| NY | NY 2019 § 364-j. Effective: December 16, 2019 | 26-b. Managed care providers shall not require prior authorization for an initial or renewal prescription for buprenorphine or injectable naltrexone for detoxification or maintenance treatment of opioid addiction unless the prescription is for a non-preferred or non-formulary form of the drug or as otherwise required by section 1927(k)(6) of the Social Security Act. | Medicaid | buprenorphine | PDL | 2019 |
| NY | NY 2018 § 273. Effective: June 22, 2016 | 10. Prior authorization shall not be required for an initial or renewal prescription for buprenorphine or injectable naltrexone for detoxification or maintenance treatment of opioid addiction unless the prescription is for a non-preferred or non-formulary form of such drug as otherwise required by section 1927(k)(6) of the Social Security Act. | Medicaid | buprenorphine | PDL | 2018 |
| NY | NY 2018 § 273. Effective: June 22, 2016 | 10. Prior authorization shall not be required for an initial or renewal prescription for buprenorphine or injectable naltrexone for detoxification or maintenance treatment of opioid addiction unless the prescription is for a non-preferred or non-formulary form of such drug as otherwise required by section 1927(k)(6) of the Social Security Act. | Medicaid | buprenorphine | PDL | 2018 |
| NY | NY 2017 § 273. Effective: June 22, 2016 | 10. Prior authorization shall not be required for an initial or renewal prescription for buprenorphine or injectable naltrexone for detoxification or maintenance treatment of opioid addiction unless the prescription is for a non-preferred or non-formulary form of such drug as otherwise required by section 1927(k)(6) of the Social Security Act. | Medicaid | buprenorphine | PDL | 2017 |
| NY | NY 2017 § 273. Effective: June 22, 2016 | 10. Prior authorization shall not be required for an initial or renewal prescription for buprenorphine or injectable naltrexone for detoxification or maintenance treatment of opioid addiction unless the prescription is for a non-preferred or non-formulary form of such drug as otherwise required by section 1927(k)(6) of the Social Security Act. | Medicaid | buprenorphine | PDL | 2017 |
| ME | ME 2019 24-A M.R.S.A. § 4304. Effective: September 19, 2019. | 2-A. Prior authorization of medication-assisted treatment for opioid use disorder. A carrier may not require prior authorization for medication-assisted treatment for opioid use disorder for the prescription of at least one drug for each therapeutic class of medication used in medication-assisted treatment, except that a carrier may not impose any prior authorization requirements on a pregnant woman for medication-assisted treatment for opioid use disorder. For the purposes of this subsection, “medication-assisted treatment” means an evidence-based practice that combines pharmacological interventions with substance use disorder counseling. | All insurers | buprenorphine (at least one) | General | 2019 |
| WA | WA 2019 48.43.760. Effective July 28, 2019. | For health plans issued or renewed on or after January 1, 2020, a health carrier shall provide coverage without prior authorization of at least one federal food and drug administration approved product for the treatment of opioid use disorder in the drug classes opioid agonists, opioid antagonists, and opioid partial agonists. | All insurers | buprenorphine (at least one) | General | 2019 |
| WA | WA 2019 74.09.645. Effective: July 28, 2019. | Upon initiation or renewal of a contract with the authority to administer a medicaid managed care plan, a managed health care system shall provide coverage without prior authorization of at least one federal food and drug administration approved product for the treatment of opioid use disorder in the drug classes opioid agonists, opioid antagonists, and opioid partial agonists. | Medicaid | buprenorphine (at least one) | General | 2019 |
| OR | O.R.S. § Ch. 583, § 7 (eff. July 23, 2019) | Ch. 583, § 7. Restrictions on prior authorization requirements for medication-assisted treatment of substance abuse disorders; rulemaking authority  (1) The Oregon Health Authority shall prohibit coordinated care organizations and public payers of health insurance, when reimbursing the cost of medication-assisted treatment for treating substance use disorders, including opioid and opiate addiction, from requiring prior authorization of payment during the first 30 days of medication-assisted treatment. (2) The authority may adopt rules to carry out this section. Credits Laws 2019, c. 583, § 7, eff. July 23, 2019, operative Jan. 1, 2020. | Medicaid | methadone | 30 days | 2019 |
| AR | AR 2019 A.C.A. § 23-99-1119 Effective: April 12, 2019 | (a) Except in the case of injectables, a healthcare insurer, including Medicaid, shall not: (1) Require prior authorization in order for a patient to obtain coverage of buprenorphine, naloxone, naltrexone, methadone, and their various formulations and combinations approved by the United States Food and Drug Administration for the treatment of opioid addiction | All insurers | methadone | General | 2019 |
| DC | DC 2019 § 31-3175.05 Effective: March 13, 2019 | Medicaid shall provide coverage for medication-assisted treatment prescribed for the treatment of substance use disorders; provided, that medication assisted treatment covered in accordance with this section shall not be subject to:   (1) Utilization control, other than those processes specified by the American Society of Addiction Medicine;   (2) Prior authorization; | Medicaid | methadone | General | 2019 |
| IL | IL 2019 215 ILCS 5/370c Effective: August 16, 2019 | (6) An issuer of a group health benefit plan may provide or offer coverage required under this Section through a managed care plan. (6.5) An individual or group health benefit plan amended, delivered, issued, or renewed on or after January 1, 2019 (the effective date of Public Act 100-1024): (A) shall not impose prior authorization requirements, other than those established under the Treatment Criteria for Addictive, Substance-Related, and Co-Occurring Conditions established by the American Society of Addiction Medicine, on a prescription medication approved by the United States Food and Drug Administration that is prescribed or administered for the treatment of substance use disorders; | All insurers | methadone | General | 2019 |
| MD | MD 2019 § 15-851 Effective: May 25, 2017 | § 15-851. Prior authorization requirement for drugs to treat opioid use disorders prohibited Currentness Scope of section (a)(1) This section applies to: (i) insurers and nonprofit health service plans that provide coverage for substance use disorder benefits or prescription drugs under individual, group, or blanket health insurance policies or contracts that are issued or delivered in the State; and (ii) health maintenance organizations that provide coverage for substance use disorder benefits or prescription drugs under individual or group contracts that are issued or delivered in the State. (2) An insurer, a nonprofit health service plan, or a health maintenance organization that provides coverage for substance use disorder benefits under the medical benefit or for prescription drugs through a pharmacy benefits manager is subject to the requirements of this section. Prohibition (b) An entity subject to this section may not apply a prior authorization requirement for a prescription drug: (1) when used for treatment of an opioid use disorder; and (2) that contains methadone, buprenorphine, or naltrexone. | All insurers | methadone | General | 2019 |
| MO | MO 2019 V.A.M.S. 191.1165 Effective: August 28, 2019 | MO 2019 V.A.M.S. 191.1165 Effective: August 28, 2019 3. MAT medications provided for in this section shall not be subject to any of the following: (1) Any annual or lifetime dollar limitations; (2) Financial requirements and quantitative treatment limitations that do not comply with the Mental Health Parity and Addiction Equity Act of 2008 (MHPAEA), specifically 45 CFR 146.136(c)(3); (3) Step therapy or other similar drug utilization strategy or policy when it conflicts or interferes with a prescribed or recommended course of treatment from a licensed health care professional; and (4) Prior authorization for MAT medications as specified in this section. 4. MAT medications outlined in this section shall apply to all health insurance plans delivered in the state of Missouri. | All insurers | methadone | General | 2019 |
| NJ | NJ 2019 30:4D-6m <Section effective on Oct. 13, 2019.> | Provision of benefits for certain drugs used in the treatment of substance use disorders; guidelines for safety and efficacy Currentness <Section effective on Oct. 13, 2019.> a. Notwithstanding any State law or regulation to the contrary, the Department of Human Services shall ensure that the provision of benefits for methadone, buprenorphine, naltrexone, combination drugs containing buprenorphine and naloxone, and, if authorized by the Commissioner of Human Services, other medications approved by the federal Food and Drug Administration for the treatment of substance use disorders, to eligible persons receiving services funded by the Division of Mental Health and Addiction Services in the Department of Human Services as well as to eligible persons under the Medicaid program, established pursuant to P.L.1968, c. 413 (C.30:4D-1 et seq.), shall be provided without the imposition of any prior authorization requirements, provided that the treatment is prescribed or administered by a licensed medical practitioner who is authorized to prescribe or administer that treatment pursuant to State and federal law. The Commissioner of Human Services may develop and issue guidelines to ensure the safety and efficacy of benefits provided pursuant to this section. b. As used in this act: “Substance use disorder” is as defined by the American Psychiatric Association in the Diagnostic and Statistical Manual of Mental Disorders, Fifth Edition and any subsequent editions and shall include substance use withdrawal. | Medicaid | methadone | General | 2019 |
| NJ | NJ 2019 1748A-7kk eff. May 16, 2017 | i. The benefits for medication-assisted treatments for substance use disorder shall be provided when determined medically necessary by the covered person's physician, psychologist or psychiatrist without the imposition of any prior authorization or other prospective utilization management requirements. | Non Medicaid | methadone | General | 2019 |
| TX | TX 2019 § 32.03115 Effective: September 1, 2019 | (a) In this section, “medication-assisted opioid or substance use disorder treatment” means the use of methadone, buprenorphine, oral buprenorphine/naloxone, or naltrexone to treat opioid or substance use disorder. (b) Notwithstanding Sections 531.072 and 531.073, Government Code, or any other law and subject to Subsections (c) and (d), the commission shall provide medical assistance reimbursement for medication-assisted opioid or substance use disorder treatment without requiring a recipient of medical assistance or health care provider to obtain prior authorization or precertification for the treatment, except as needed to minimize the opportunity for fraud, waste, or abuse.1 (c) The duty to provide medical assistance reimbursement for medication-assisted opioid or substance use disorder treatment under Subsection (b) does not apply with respect to: (1) a prescription for methadone; | Medicaid | methadone | General | 2019 |
| IL | IL 2018 305 ILCS 5/5-5 Effective: January 1, 2018 | Notwithstanding any other provision of this Code to the contrary, on or after July 1, 2015, all FDA approved forms of medication assisted treatment prescribed for the treatment of alcohol dependence or treatment of opioid dependence shall be covered under both fee for service and managed care medical assistance programs for persons who are otherwise eligible for medical assistance under this Article and shall not be subject to any (1) utilization control, other than those established under the American Society of Addiction Medicine patient placement criteria, (2) prior authorization mandate, or (3) lifetime restriction limit mandate. On or after July 1, 2015, opioid antagonists prescribed for the treatment of an opioid overdose, including the medication product, administration devices, and any pharmacy fees related to the dispensing and administration of the opioid antagonist, shall be covered under the medical assistance program for persons who are otherwise eligible for medical assistance under this Article. As used in this Section, “opioid antagonist” means a drug that binds to opioid receptors and blocks or inhibits the effect of opioids acting on those receptors, including, but not limited to, naloxone hydrochloride or any other similarly acting drug approved by the U.S. Food and Drug Administration. | Medicaid | methadone | General | 2018 |
| MD | MD 2018 § 15-851 Effective: May 25, 2017 | § 15-851. Prior authorization requirement for drugs to treat opioid use disorders prohibited Currentness Scope of section (a)(1) This section applies to: (i) insurers and nonprofit health service plans that provide coverage for substance use disorder benefits or prescription drugs under individual, group, or blanket health insurance policies or contracts that are issued or delivered in the State; and (ii) health maintenance organizations that provide coverage for substance use disorder benefits or prescription drugs under individual or group contracts that are issued or delivered in the State. (2) An insurer, a nonprofit health service plan, or a health maintenance organization that provides coverage for substance use disorder benefits under the medical benefit or for prescription drugs through a pharmacy benefits manager is subject to the requirements of this section. Prohibition (b) An entity subject to this section may not apply a prior authorization requirement for a prescription drug: (1) when used for treatment of an opioid use disorder; and (2) that contains methadone, buprenorphine, or naltrexone. Credits Added by Acts 2017, c. 581, § 1, eff. May 25, 2017. | All insurers | methadone | General | 2018 |
| NJ | NJ 2018 17:48A-7kk eff. May 16, 2017 | i. The benefits for medication-assisted treatments for substance use disorder shall be provided when determined medically necessary by the covered person's physician, psychologist or psychiatrist without the imposition of any prior authorization or other prospective utilization management requirements. | NonMedicaid | methadone | General | 2018 |
| IL | IL 2017 305 ILCS 5/5-5 Effective: January 1, 2017 | Notwithstanding any other provision of this Code to the contrary, on or after July 1, 2015, all FDA approved forms of medication assisted treatment prescribed for the treatment of alcohol dependence or treatment of opioid dependence shall be covered under both fee for service and managed care medical assistance programs for persons who are otherwise eligible for medical assistance under this Article and shall not be subject to any (1) utilization control, other than those established under the American Society of Addiction Medicine patient placement criteria, (2) prior authorization mandate, or (3) lifetime restriction limit mandate. On or after July 1, 2015, opioid antagonists prescribed for the treatment of an opioid overdose, including the medication product, administration devices, and any pharmacy fees related to the dispensing and administration of the opioid antagonist, shall be covered under the medical assistance program for persons who are otherwise eligible for medical assistance under this Article. As used in this Section, “opioid antagonist” means a drug that binds to opioid receptors and blocks or inhibits the effect of opioids acting on those receptors, including, but not limited to, naloxone hydrochloride or any other similarly acting drug approved by the U.S. Food and Drug Administration. | Medicaid | methadone | General | 2017 |
| MD | MD 2017 § 15-851 Effective: May 25, 2017 | MD Code, Insurance, § 15-851 § 15-851. Prior authorization requirement for drugs to treat opioid use disorders prohibited Currentness Scope of section (a)(1) This section applies to: (i) insurers and nonprofit health service plans that provide coverage for substance use disorder benefits or prescription drugs under individual, group, or blanket health insurance policies or contracts that are issued or delivered in the State; and (ii) health maintenance organizations that provide coverage for substance use disorder benefits or prescription drugs under individual or group contracts that are issued or delivered in the State. (2) An insurer, a nonprofit health service plan, or a health maintenance organization that provides coverage for substance use disorder benefits under the medical benefit or for prescription drugs through a pharmacy benefits manager is subject to the requirements of this section. Prohibition (b) An entity subject to this section may not apply a prior authorization requirement for a prescription drug: (1) when used for treatment of an opioid use disorder; and (2) that contains methadone, buprenorphine, or naltrexone. | All insurers | methadone | General | 2017 |
| NJ | NJ 2017 17:48A-7kk Effective on May 16, 2017 | i. The benefits for medication-assisted treatments for substance use disorder shall be provided when determined medically necessary by the covered person's physician, psychologist or psychiatrist without the imposition of any prior authorization or other prospective utilization management requirements. | NonMedicaid | methadone | General | 2017 |
| IL | IL 2016 305 ILCS 5/5-5 Effective: July 1, 2016 | Notwithstanding any other provision of this Code to the contrary, on or after July 1, 2015, all FDA approved forms of medication assisted treatment prescribed for the treatment of alcohol dependence or treatment of opioid dependence shall be covered under both fee for service and managed care medical assistance programs for persons who are otherwise eligible for medical assistance under this Article and shall not be subject to any (1) utilization control, other than those established under the American Society of Addiction Medicine patient placement criteria, (2) prior authorization mandate, or (3) lifetime restriction limit mandate.  2nd law: IL 2016 305 ILCS 5/5-5 September 9, 2015 to June 30, 2016 Notwithstanding any other provision of this Code to the contrary, on or after July 1, 2015, all FDA approved forms of medication assisted treatment prescribed for the treatment of alcohol dependence or treatment of opioid dependence shall be covered under both fee for service and managed care medical assistance programs for persons who are otherwise eligible for medical assistance under this Article and shall not be subject to any (1) utilization control, other than those established under the American Society of Addiction Medicine patient placement criteria, (2) prior authorization mandate, or (3) lifetime restriction limit mandate. On or after July 1, 2015, opioid antagonists prescribed for the treatment of an opioid overdose, including the medication product, administration devices, and any pharmacy fees related to the dispensing and administration of the opioid antagonist, shall be covered under the medical assistance program for persons who are otherwise eligible for medical assistance under this Article. As used in this Section, “opioid antagonist” means a drug that binds to opioid receptors and blocks or inhibits the effect of opioids acting on those receptors, including, but not limited to, naloxone hydrochloride or any other similarly acting drug approved by the U.S. Food and Drug Administration. | Medicaid | methadone | General | 2016 |
| DE | DE 2019 § 3343. eff. Aug. 29, 2018. | (2) a. A health benefit plan that provides coverage for prescription drugs must provide coverage for the treatment of serious mental illnesses and drug and alcohol dependencies that includes immediate access, without prior authorization, to a 5-day emergency supply of prescribed medications covered under the health benefit plan for the medically necessary treatment of serious mental illnesses and drug and alcohol dependencies where an emergency medical condition, as defined in § 3349(e) of this title, exists, including a prescribed drug or medication associated with the management of opioid withdrawal or stabilization, except where otherwise prohibited by law.  DE 2019 § 3578. eff. Sept. 29, 2017.  (2) a. A health benefit plan that provides coverage for prescription drugs must provide coverage for the treatment of serious mental illnesses and drug and alcohol dependencies that include immediate access, without prior authorization, to a 5 day emergency supply of prescribed medications covered under the health benefit plan for the medically necessary treatment of serious mental illnesses and drug and alcohol dependencies where an emergency medical condition, as defined in § 3565(e) of this title, exists, including a prescribed drug or medication associated with the management of opioid withdrawal or stabilization, except where otherwise prohibited by law. | All insurers | methadone | If on formulary, 5 days | 2019 |
| DE | DE 2018 § 3343. eff. Aug. 29, 2018. | (2) a. A health benefit plan that provides coverage for prescription drugs must provide coverage for the treatment of serious mental illnesses and drug and alcohol dependencies that includes immediate access, without prior authorization, to a 5-day emergency supply of prescribed medications covered under the health benefit plan for the medically necessary treatment of serious mental illnesses and drug and alcohol dependencies where an emergency medical condition, as defined in § 3349(e) of this title, exists, including a prescribed drug or medication associated with the management of opioid withdrawal or stabilization, except where otherwise prohibited by law.  DE 2018 § 3578. eff. Sept. 29, 2017.  (2) a. A health benefit plan that provides coverage for prescription drugs must provide coverage for the treatment of serious mental illnesses and drug and alcohol dependencies that include immediate access, without prior authorization, to a 5 day emergency supply of prescribed medications covered under the health benefit plan for the medically necessary treatment of serious mental illnesses and drug and alcohol dependencies where an emergency medical condition, as defined in § 3565(e) of this title, exists, including a prescribed drug or medication associated with the management of opioid withdrawal or stabilization, except where otherwise prohibited by law. | All insurers | methadone | If on formulary, 5 days | 2018 |
| AR | AR 2019 A.C.A. § 23-99-1119 - Effective: April 12, 2019. | (a) Except in the case of injectables, a healthcare insurer, including Medicaid, shall not: (1) Require prior authorization in order for a patient to obtain coverage of buprenorphine, naloxone, naltrexone, methadone, and their various formulations and combinations approved by the United States Food and Drug Administration for the treatment of opioid addiction; or (2) Impose any other requirement other than a valid prescription and compliance with the medication-assisted treatment guidelines issued by the Substance Abuse and Mental Health Services Administration under the United States Department of Health and Human Services in order for a patient to obtain coverage for buprenorphine, naloxone, naltrexone, methadone, and their various formulations and combinations approved by the United States Food and Drug Administration for the treatment of opioid addiction. (b) Subdivision (a)(1) of this section shall only apply to the Arkansas Medicaid Program as it pertains to prescription drugs for treatment of opioid addiction designated as preferred on the evidence-based preferred drug list provided there is at least one (1) of each of the drugs listed in subdivision (a)(1) of this section with the preferred designation on the preferred drug list or available without prior authorization. | Medicaid | methadone | PDL | 2019 |
| ME | ME 2019 24-A M.R.S.A. § 4304. Effective: September 19, 2019. | 2-A. Prior authorization of medication-assisted treatment for opioid use disorder. A carrier may not require prior authorization for medication-assisted treatment for opioid use disorder for the prescription of at least one drug for each therapeutic class of medication used in medication-assisted treatment, except that a carrier may not impose any prior authorization requirements on a pregnant woman for medication-assisted treatment for opioid use disorder. For the purposes of this subsection, “medication-assisted treatment” means an evidence-based practice that combines pharmacological interventions with substance use disorder counseling. | All insurers | methadone (at least one) | General | 2019 |
| WA | WA 2019 48.43.760. Effective July 28, 2019. | For health plans issued or renewed on or after January 1, 2020, a health carrier shall provide coverage without prior authorization of at least one federal food and drug administration approved product for the treatment of opioid use disorder in the drug classes opioid agonists, opioid antagonists, and opioid partial agonists. | All insurers | methadone (at least one) | General | 2019 |
| WA | WA 2019 74.09.645. Effective: July 28, 2019. | Upon initiation or renewal of a contract with the authority to administer a medicaid managed care plan, a managed health care system shall provide coverage without prior authorization of at least one federal food and drug administration approved product for the treatment of opioid use disorder in the drug classes opioid agonists, opioid antagonists, and opioid partial agonists. | Medicaid | methadone (at least one) | General | 2019 |
| OR | O.R.S. § Ch. 583, § 7 (eff. July 23, 2019) | Ch. 583, § 7. Restrictions on prior authorization requirements for medication-assisted treatment of substance abuse disorders; rulemaking authority  (1) The Oregon Health Authority shall prohibit coordinated care organizations and public payers of health insurance, when reimbursing the cost of medication-assisted treatment for treating substance use disorders, including opioid and opiate addiction, from requiring prior authorization of payment during the first 30 days of medication-assisted treatment. (2) The authority may adopt rules to carry out this section. Credits Laws 2019, c. 583, § 7, eff. July 23, 2019, operative Jan. 1, 2020. | Medicaid | naltrexone | 30 days | 2019 |
| AR | AR 2019 A.C.A. § 23-99-1119 Effective: April 12, 2019. | (a) Except in the case of injectables, a healthcare insurer, including Medicaid, shall not: (1) Require prior authorization in order for a patient to obtain coverage of buprenorphine, naloxone, naltrexone , methadone, and their various formulations and combinations approved by the United States Food and Drug Administration for the treatment of opioid addiction; | All insurers | naltrexone | General | 2019 |
| DC | DC 2019 § 31-3175.05 Effective: March 13, 2019 | Medicaid shall provide coverage for medication-assisted treatment prescribed for the treatment of substance use disorders; provided, that medication assisted treatment covered in accordance with this section shall not be subject to:   (1) Utilization control, other than those processes specified by the American Society of Addiction Medicine;   (2) Prior authorization; | Medicaid | naltrexone | General | 2019 |
| IL | IL 2019 215 ILCS 5/370c Effective: August 16, 2019 | (6) An issuer of a group health benefit plan may provide or offer coverage required under this Section through a managed care plan. (6.5) An individual or group health benefit plan amended, delivered, issued, or renewed on or after January 1, 2019 (the effective date of Public Act 100-1024): (A) shall not impose prior authorization requirements, other than those established under the Treatment Criteria for Addictive, Substance-Related, and Co-Occurring Conditions established by the American Society of Addiction Medicine, on a prescription medication approved by the United States Food and Drug Administration that is prescribed or administered for the treatment of substance use disorders; | All insurers | naltrexone | General | 2019 |
| MD | MD 2019 § 15-851 Effective: May 25, 2017 | § 15-851. Prior authorization requirement for drugs to treat opioid use disorders prohibited Currentness Scope of section (a)(1) This section applies to: (i) insurers and nonprofit health service plans that provide coverage for substance use disorder benefits or prescription drugs under individual, group, or blanket health insurance policies or contracts that are issued or delivered in the State; and (ii) health maintenance organizations that provide coverage for substance use disorder benefits or prescription drugs under individual or group contracts that are issued or delivered in the State. (2) An insurer, a nonprofit health service plan, or a health maintenance organization that provides coverage for substance use disorder benefits under the medical benefit or for prescription drugs through a pharmacy benefits manager is subject to the requirements of this section. Prohibition (b) An entity subject to this section may not apply a prior authorization requirement for a prescription drug: (1) when used for treatment of an opioid use disorder; and (2) that contains methadone, buprenorphine, or naltrexone. | All insurers | naltrexone | General | 2019 |
| MO | MO 2019 V.A.M.S. 191.1165 Effective: August 28, 2019 | MO 2019 V.A.M.S. 191.1165 Effective: August 28, 2019 3. MAT medications provided for in this section shall not be subject to any of the following: (1) Any annual or lifetime dollar limitations; (2) Financial requirements and quantitative treatment limitations that do not comply with the Mental Health Parity and Addiction Equity Act of 2008 (MHPAEA), specifically 45 CFR 146.136(c)(3); (3) Step therapy or other similar drug utilization strategy or policy when it conflicts or interferes with a prescribed or recommended course of treatment from a licensed health care professional; and (4) Prior authorization for MAT medications as specified in this section. 4. MAT medications outlined in this section shall apply to all health insurance plans delivered in the state of Missouri. | All insurers | naltrexone | General | 2019 |
| NJ | NJ 2019 30:4D-6m <Section effective on Oct. 13, 2019.> | Provision of benefits for certain drugs used in the treatment of substance use disorders; guidelines for safety and efficacy Currentness <Section effective on Oct. 13, 2019.> a. Notwithstanding any State law or regulation to the contrary, the Department of Human Services shall ensure that the provision of benefits for methadone, buprenorphine, naltrexone, combination drugs containing buprenorphine and naloxone, and, if authorized by the Commissioner of Human Services, other medications approved by the federal Food and Drug Administration for the treatment of substance use disorders, to eligible persons receiving services funded by the Division of Mental Health and Addiction Services in the Department of Human Services as well as to eligible persons under the Medicaid program, established pursuant to P.L.1968, c. 413 (C.30:4D-1 et seq.), shall be provided without the imposition of any prior authorization requirements, provided that the treatment is prescribed or administered by a licensed medical practitioner who is authorized to prescribe or administer that treatment pursuant to State and federal law. The Commissioner of Human Services may develop and issue guidelines to ensure the safety and efficacy of benefits provided pursuant to this section. b. As used in this act: “Substance use disorder” is as defined by the American Psychiatric Association in the Diagnostic and Statistical Manual of Mental Disorders, Fifth Edition and any subsequent editions and shall include substance use withdrawal. | Medicaid | naltrexone | General | 2019 |
| NJ | NJ 2019 1748A-7kk eff. May 16, 2017 | i. The benefits for medication-assisted treatments for substance use disorder shall be provided when determined medically necessary by the covered person's physician, psychologist or psychiatrist without the imposition of any prior authorization or other prospective utilization management requirements. | Non Medicaid | naltrexone | General | 2019 |
| TX | TX 2019 § 32.03115 Effective: September 1, 2019 | (a) In this section, “medication-assisted opioid or substance use disorder treatment” means the use of methadone, buprenorphine, oral buprenorphine/naloxone, or naltrexone to treat opioid or substance use disorder. (b) Notwithstanding Sections 531.072 and 531.073, Government Code, or any other law and subject to Subsections (c) and (d), the commission shall provide medical assistance reimbursement for medication-assisted opioid or substance use disorder treatment without requiring a recipient of medical assistance or health care provider to obtain prior authorization or precertification for the treatment, except as needed to minimize the opportunity for fraud, waste, or abuse.1 (c) The duty to provide medical assistance reimbursement for medication-assisted opioid or substance use disorder treatment under Subsection (b) does not apply with respect to: (1) a prescription for methadone; | Medicaid | naltrexone | General | 2019 |
| IL | IL 2018 305 ILCS 5/5-5 Effective: January 1, 2018 | Notwithstanding any other provision of this Code to the contrary, on or after July 1, 2015, all FDA approved forms of medication assisted treatment prescribed for the treatment of alcohol dependence or treatment of opioid dependence shall be covered under both fee for service and managed care medical assistance programs for persons who are otherwise eligible for medical assistance under this Article and shall not be subject to any (1) utilization control, other than those established under the American Society of Addiction Medicine patient placement criteria, (2) prior authorization mandate, or (3) lifetime restriction limit mandate. On or after July 1, 2015, opioid antagonists prescribed for the treatment of an opioid overdose, including the medication product, administration devices, and any pharmacy fees related to the dispensing and administration of the opioid antagonist, shall be covered under the medical assistance program for persons who are otherwise eligible for medical assistance under this Article. As used in this Section, “opioid antagonist” means a drug that binds to opioid receptors and blocks or inhibits the effect of opioids acting on those receptors, including, but not limited to, naloxone hydrochloride or any other similarly acting drug approved by the U.S. Food and Drug Administration. | Medicaid | naltrexone | General | 2018 |
| MD | MD 2018 § 15-851 Effective: May 25, 2017 | § 15-851. Prior authorization requirement for drugs to treat opioid use disorders prohibited Currentness Scope of section (a)(1) This section applies to: (i) insurers and nonprofit health service plans that provide coverage for substance use disorder benefits or prescription drugs under individual, group, or blanket health insurance policies or contracts that are issued or delivered in the State; and (ii) health maintenance organizations that provide coverage for substance use disorder benefits or prescription drugs under individual or group contracts that are issued or delivered in the State. (2) An insurer, a nonprofit health service plan, or a health maintenance organization that provides coverage for substance use disorder benefits under the medical benefit or for prescription drugs through a pharmacy benefits manager is subject to the requirements of this section. Prohibition (b) An entity subject to this section may not apply a prior authorization requirement for a prescription drug: (1) when used for treatment of an opioid use disorder; and (2) that contains methadone, buprenorphine, or naltrexone. Credits Added by Acts 2017, c. 581, § 1, eff. May 25, 2017. | All insurers | naltrexone | General | 2018 |
| NJ | NJ 2018 17:48A-7kk eff. May 16, 2017 | i. The benefits for medication-assisted treatments for substance use disorder shall be provided when determined medically necessary by the covered person's physician, psychologist or psychiatrist without the imposition of any prior authorization or other prospective utilization management requirements. | NonMedicaid | naltrexone | General | 2018 |
| IL | IL 2017 305 ILCS 5/5-5 Effective: January 1, 2017 | Notwithstanding any other provision of this Code to the contrary, on or after July 1, 2015, all FDA approved forms of medication assisted treatment prescribed for the treatment of alcohol dependence or treatment of opioid dependence shall be covered under both fee for service and managed care medical assistance programs for persons who are otherwise eligible for medical assistance under this Article and shall not be subject to any (1) utilization control, other than those established under the American Society of Addiction Medicine patient placement criteria, (2) prior authorization mandate, or (3) lifetime restriction limit mandate. On or after July 1, 2015, opioid antagonists prescribed for the treatment of an opioid overdose, including the medication product, administration devices, and any pharmacy fees related to the dispensing and administration of the opioid antagonist, shall be covered under the medical assistance program for persons who are otherwise eligible for medical assistance under this Article. As used in this Section, “opioid antagonist” means a drug that binds to opioid receptors and blocks or inhibits the effect of opioids acting on those receptors, including, but not limited to, naloxone hydrochloride or any other similarly acting drug approved by the U.S. Food and Drug Administration. | Medicaid | naltrexone | General | 2017 |
| MD | MD 2017 § 15-851 Effective: May 25, 2017 | MD Code, Insurance, § 15-851 § 15-851. Prior authorization requirement for drugs to treat opioid use disorders prohibited Currentness Scope of section (a)(1) This section applies to: (i) insurers and nonprofit health service plans that provide coverage for substance use disorder benefits or prescription drugs under individual, group, or blanket health insurance policies or contracts that are issued or delivered in the State; and (ii) health maintenance organizations that provide coverage for substance use disorder benefits or prescription drugs under individual or group contracts that are issued or delivered in the State. (2) An insurer, a nonprofit health service plan, or a health maintenance organization that provides coverage for substance use disorder benefits under the medical benefit or for prescription drugs through a pharmacy benefits manager is subject to the requirements of this section. Prohibition (b) An entity subject to this section may not apply a prior authorization requirement for a prescription drug: (1) when used for treatment of an opioid use disorder; and (2) that contains methadone, buprenorphine, or naltrexone. | All insurers | naltrexone | General | 2017 |
| NJ | NJ 2017 17:48A-7kk Effective on May 16, 2017 | i. The benefits for medication-assisted treatments for substance use disorder shall be provided when determined medically necessary by the covered person's physician, psychologist or psychiatrist without the imposition of any prior authorization or other prospective utilization management requirements. | Non Medicaid | naltrexone | General | 2017 |
| IL | IL 2016 305 ILCS 5/5-5 Effective: July 1, 2016 | Notwithstanding any other provision of this Code to the contrary, on or after July 1, 2015, all FDA approved forms of medication assisted treatment prescribed for the treatment of alcohol dependence or treatment of opioid dependence shall be covered under both fee for service and managed care medical assistance programs for persons who are otherwise eligible for medical assistance under this Article and shall not be subject to any (1) utilization control, other than those established under the American Society of Addiction Medicine patient placement criteria, (2) prior authorization mandate, or (3) lifetime restriction limit mandate.  2nd law: IL 2016 305 ILCS 5/5-5 September 9, 2015 to June 30, 2016 Notwithstanding any other provision of this Code to the contrary, on or after July 1, 2015, all FDA approved forms of medication assisted treatment prescribed for the treatment of alcohol dependence or treatment of opioid dependence shall be covered under both fee for service and managed care medical assistance programs for persons who are otherwise eligible for medical assistance under this Article and shall not be subject to any (1) utilization control, other than those established under the American Society of Addiction Medicine patient placement criteria, (2) prior authorization mandate, or (3) lifetime restriction limit mandate. On or after July 1, 2015, opioid antagonists prescribed for the treatment of an opioid overdose, including the medication product, administration devices, and any pharmacy fees related to the dispensing and administration of the opioid antagonist, shall be covered under the medical assistance program for persons who are otherwise eligible for medical assistance under this Article. As used in this Section, “opioid antagonist” means a drug that binds to opioid receptors and blocks or inhibits the effect of opioids acting on those receptors, including, but not limited to, naloxone hydrochloride or any other similarly acting drug approved by the U.S. Food and Drug Administration. | Medicaid | naltrexone | General | 2016 |
| NY | NY 2019 § 273. Effective: June 22, 2016. | 10. Prior authorization shall not be required for an initial or renewal prescription for buprenorphine or injectable naltrexone for detoxification or maintenance treatment of opioid addiction unless the prescription is for a non-preferred or non-formulary form of such drug as otherwise required by section 1927(k)(6) of the Social Security Act. | Medicaid | naltrexone | If on formulary | 2019 |
| NY | NY 2019 § 364-j. Effective: December 16, 2019. | 26-b. Managed care providers shall not require prior authorization for an initial or renewal prescription for buprenorphine or injectable naltrexone for detoxification or maintenance treatment of opioid addiction unless the prescription is for a non-preferred or non-formulary form of the drug or as otherwise required by section 1927(k)(6) of the Social Security Act. | Medicaid | naltrexone | If on formulary | 2019 |
| NY | NY 2018 § 364-j. Effective: April 20, 2017. | 26-b. Managed care providers shall not require prior authorization for an initial or renewal prescription for buprenorphine or injectable naltrexone for detoxification or maintenance treatment of opioid addiction unless the prescription is for a non-preferred or non-formulary form of the drug or as otherwise required by section 1927(k)(6) of the Social Security Act. | Medicaid | naltrexone | If on formulary | 2018 |
| NY | NY 2018 § 364-j. Effective: April 20, 2017. | 26-b. Managed care providers shall not require prior authorization for an initial or renewal prescription for buprenorphine or injectable naltrexone for detoxification or maintenance treatment of opioid addiction unless the prescription is for a non-preferred or non-formulary form of the drug or as otherwise required by section 1927(k)(6) of the Social Security Act. | Medicaid | naltrexone | If on formulary | 2018 |
| NY | NY 2017 § 364-j. Effective: June 22, 2016. | 26-b. Managed care providers shall not require prior authorization for an initial or renewal prescription for buprenorphine or injectable naltrexone for detoxification or maintenance treatment of opioid addiction unless the prescription is for a non-preferred or non-formulary form of the drug or as otherwise required by section 1927(k)(6) of the Social Security Act. | Medicaid | naltrexone | If on formulary | 2017 |
| NY | NY 2017 § 364-j. Effective: June 22, 2016. | 26-b. Managed care providers shall not require prior authorization for an initial or renewal prescription for buprenorphine or injectable naltrexone for detoxification or maintenance treatment of opioid addiction unless the prescription is for a non-preferred or non-formulary form of the drug or as otherwise required by section 1927(k)(6) of the Social Security Act. | Medicaid | naltrexone | If on formulary | 2017 |
| DE | DE 2019 § 3343. eff. Aug. 29, 2018. | (2) a. A health benefit plan that provides coverage for prescription drugs must provide coverage for the treatment of serious mental illnesses and drug and alcohol dependencies that includes immediate access, without prior authorization, to a 5-day emergency supply of prescribed medications covered under the health benefit plan for the medically necessary treatment of serious mental illnesses and drug and alcohol dependencies where an emergency medical condition, as defined in § 3349(e) of this title, exists, including a prescribed drug or medication associated with the management of opioid withdrawal or stabilization, except where otherwise prohibited by law.  DE 2019 § 3578. eff. Sept. 29, 2017.  (2) a. A health benefit plan that provides coverage for prescription drugs must provide coverage for the treatment of serious mental illnesses and drug and alcohol dependencies that include immediate access, without prior authorization, to a 5 day emergency supply of prescribed medications covered under the health benefit plan for the medically necessary treatment of serious mental illnesses and drug and alcohol dependencies where an emergency medical condition, as defined in § 3565(e) of this title, exists, including a prescribed drug or medication associated with the management of opioid withdrawal or stabilization, except where otherwise prohibited by law. | All insurers | naltrexone | If on formulary, 5 days | 2019 |
| DE | DE 2018 § 3343. eff. Aug. 29, 2018. | (2) a. A health benefit plan that provides coverage for prescription drugs must provide coverage for the treatment of serious mental illnesses and drug and alcohol dependencies that includes immediate access, without prior authorization, to a 5-day emergency supply of prescribed medications covered under the health benefit plan for the medically necessary treatment of serious mental illnesses and drug and alcohol dependencies where an emergency medical condition, as defined in § 3349(e) of this title, exists, including a prescribed drug or medication associated with the management of opioid withdrawal or stabilization, except where otherwise prohibited by law.  DE 2018 § 3578. eff. Sept. 29, 2017.  (2) a. A health benefit plan that provides coverage for prescription drugs must provide coverage for the treatment of serious mental illnesses and drug and alcohol dependencies that include immediate access, without prior authorization, to a 5 day emergency supply of prescribed medications covered under the health benefit plan for the medically necessary treatment of serious mental illnesses and drug and alcohol dependencies where an emergency medical condition, as defined in § 3565(e) of this title, exists, including a prescribed drug or medication associated with the management of opioid withdrawal or stabilization, except where otherwise prohibited by law. | All insurers | naltrexone | If on formulary, 5 days | 2018 |
| AR | AR 2019 A.C.A. § 23-99-1119 Effective: April 12, 2019. | (a) Except in the case of injectables, a healthcare insurer, including Medicaid, shall not: (1) Require prior authorization in order for a patient to obtain coverage of buprenorphine, naloxone, naltrexone , methadone, and their various formulations and combinations approved by the United States Food and Drug Administration for the treatment of opioid addiction; or ... (b) Subdivision (a)(1) of this section shall only apply to the Arkansas Medicaid Program as it pertains to prescription drugs for treatment of opioid addiction designated as preferred on the evidence-based preferred drug list provided there is at least one (1) of each of the drugs listed in subdivision (a)(1) of this section with the preferred designation on the preferred drug list or available without prior authorization. | Medicaid | naltrexone | PDL | 2019 |
| NY | NY 2019 § 273. Effective: June 22, 2016 | 10. Prior authorization shall not be required for an initial or renewal prescription for buprenorphine or injectable naltrexone for detoxification or maintenance treatment of opioid addiction unless the prescription is for a non-preferred or non-formulary form of such drug as otherwise required by section 1927(k)(6) of the Social Security Act. | Medicaid | naltrexone | PDL | 2019 |
| NY | NY 2019 § 364-j. Effective: December 16, 2019 | 26-b. Managed care providers shall not require prior authorization for an initial or renewal prescription for buprenorphine or injectable naltrexone for detoxification or maintenance treatment of opioid addiction unless the prescription is for a non-preferred or non-formulary form of the drug or as otherwise required by section 1927(k)(6) of the Social Security Act. | Medicaid | naltrexone | PDL | 2019 |
| NY | NY 2018 § 364-j. Effective: April 20, 2017 | 26-b. Managed care providers shall not require prior authorization for an initial or renewal prescription for buprenorphine or injectable naltrexone for detoxification or maintenance treatment of opioid addiction unless the prescription is for a non-preferred or non-formulary form of the drug or as otherwise required by section 1927(k)(6) of the Social Security Act. | Medicaid | naltrexone | PDL | 2018 |
| NY | NY 2018 § 364-j. Effective: April 20, 2017 | 26-b. Managed care providers shall not require prior authorization for an initial or renewal prescription for buprenorphine or injectable naltrexone for detoxification or maintenance treatment of opioid addiction unless the prescription is for a non-preferred or non-formulary form of the drug or as otherwise required by section 1927(k)(6) of the Social Security Act. | Medicaid | naltrexone | PDL | 2018 |
| NY | NY 2017 § 364-j. Effective: June 22, 2016 | 26-b. Managed care providers shall not require prior authorization for an initial or renewal prescription for buprenorphine or injectable naltrexone for detoxification or maintenance treatment of opioid addiction unless the prescription is for a non-preferred or non-formulary form of the drug or as otherwise required by section 1927(k)(6) of the Social Security Act. | Medicaid | naltrexone | PDL | 2017 |
| NY | NY 2017 § 364-j. Effective: June 22, 2016 | 26-b. Managed care providers shall not require prior authorization for an initial or renewal prescription for buprenorphine or injectable naltrexone for detoxification or maintenance treatment of opioid addiction unless the prescription is for a non-preferred or non-formulary form of the drug or as otherwise required by section 1927(k)(6) of the Social Security Act. | Medicaid | naltrexone | PDL | 2017 |
| ME | ME 2019 24-A M.R.S.A. § 4304. Effective: September 19, 2019. | 2-A. Prior authorization of medication-assisted treatment for opioid use disorder. A carrier may not require prior authorization for medication-assisted treatment for opioid use disorder for the prescription of at least one drug for each therapeutic class of medication used in medication-assisted treatment, except that a carrier may not impose any prior authorization requirements on a pregnant woman for medication-assisted treatment for opioid use disorder. For the purposes of this subsection, “medication-assisted treatment” means an evidence-based practice that combines pharmacological interventions with substance use disorder counseling. | All insurers | naltrexone (at least one) | General | 2019 |
| WA | WA 2019 48.43.760. Effective July 28, 2019. | For health plans issued or renewed on or after January 1, 2020, a health carrier shall provide coverage without prior authorization of at least one federal food and drug administration approved product for the treatment of opioid use disorder in the drug classes opioid agonists, opioid antagonists, and opioid partial agonists. | All insurers | naltrexone (at least one) | General | 2019 |
| WA | WA 2019 74.09.645. Effective: July 28, 2019. | Upon initiation or renewal of a contract with the authority to administer a medicaid managed care plan, a managed health care system shall provide coverage without prior authorization of at least one federal food and drug administration approved product for the treatment of opioid use disorder in the drug classes opioid agonists, opioid antagonists, and opioid partial agonists. | Medicaid | naltrexone (at least one) | General | 2019 |
